# Fatal viscerotropic and neurotropic disease after yellow fever vaccine: a rare manifestation leading to diagnosis of severe combined immunodeficiency in an infant

**DOI:** 10.1590/S1678-9946202466050

**Published:** 2024-08-26

**Authors:** Lara Jhullian Tolentino Vieira, Gabriela Assunção Goebel, Yuri Barcelos, Luciana Oliveira Cunha, Luisa Teles Melo Santos, Roberta Maia de Castro Romanelli, Fernanda Gontijo Minafra, Andrea Lucchesi de Carvalho, Luiz Fernando Andrade de Carvalho, Lilian Martins Oliveira Diniz

**Affiliations:** 1 Fundação Hospitalar do Estado de Minas Gerais, Hospital Infantil João Paulo II, Programa de Residência Médica em Pediatria, Belo Horizonte, Minas Gerais, Brazil; 2 Universidade Federal de Minas Gerais, Departamento de Pediatria, Belo Horizonte, Minas Gerais, Brazil

**Keywords:** Immunodeficiency, Vaccine, Yellow fever

## Abstract

Yellow fever vaccine (YFV) is a live attenuated vaccine that can cause a mild infection in immunocompetent patients. However, it may not be self-limiting in patients with inborn errors of immunity (IEI) and may be the first and most severe presentation in these patients. A 10-month-old female infant sought emergency care presenting fever for three days and diffuse exanthema. She was a previous healthy child of consanguineous parents. The child had received YFV 28 days before the onset of symptoms. Upon hospital admission, petechial rash on the limbs and hepatosplenomegaly were noted on physical exam. Laboratory tests showed thrombocytopenia, increased serum aminotransferases and elevated gamma-glutamyl transferase (GGT) and alkaline phosphatase levels. During hospitalization she developed hypoactivity, drowsiness, and hypotonia. The possibility of viscerotropic and neurotropic vaccine associated disease was suspected and a possible primary immunodeficiency disease considered. The patient was tested for antibodies against the yellow fever virus (MAC ELISA) on serum and cerebrospinal fluid (CSF) samples, showing positive IgM results. Immunophenotyping showed low levels of lymphocytes and absence of T-cell receptor excision circles (TREC), leading to diagnose of severe combined immunodeficiency disease (SCID). Despite treatment, after 35 days of hospitalization, she evolved to cardiorespiratory arrest and death. Serious adverse events after administration of the YFV are rare and associated with neurological or visceral involvement in most cases. The unfavorable outcome highlights the importance of neonatal screening for SCID and the clinical suspicion of primary immunodeficiencies in infants who have serious adverse events to live virus vaccines.

## INTRODUCTION

Primary immunodeficiencies are a heterogeneous group of inherited pathologies that affect the immune system. Severe combined immunodeficiency (SCID) is a rare disease that affects approximately 1: 55,000 newborns and results from various monogenic defects that impair immune function and brings on early severe and life-threatening infections. The main stay of treatment for SCID is hematopoietic stem cell transplant (HSCT). Although overall rare, it constitutes a major burden on affected children, their families, and the health system, especially in communities with a high rate of consanguinity, where incidence and prevalence of recessive inborn errors of immunity (IEI) are expected to be high^1^.

Patients with SCID have a very low number of T cells and dysfunctional B lymphocytes, characterizing a combined cellular and humoral immunodeficiency, making them susceptible to infections. Clinical manifestations usually begin in the first six months of life with severe and persistent infections caused by any type of microorganism, as well as growth deficit and chronic diarrhea. Laboratory tests have found the presence of lymphopenia, reduced T lymphocytes (CD3+, CD4+, and CD8+), with or without a decrease in the number of B lymphocytes (CD19 +) and/or natural killer cells (CD16+, CD56+).^2^ In the ideal scenario, patients with SCID are diagnosed via newborn screening. However, in Brazil, where disease screening is not widely available, the diagnosis is obtained only after onset of signs and symptoms.

For these patients, no live virus or bacteria vaccines should be administered, not only to prevent inadequate responses, but also due to the risk of causing serious disseminated diseases secondary to vaccine microorganism^2^. In this context, the literature describes several cases of infants without an early SCID diagnosis who were vaccinated with the Bacillus Calmette-Guerin (BCG) vaccine and developed the vaccine-associated disease. BCG vaccine is a live attenuated vaccine form of *Mycobacterium bovis* that is often administrated to infants in Brazil soon after birth. BCG-related disease is a well-known manifestation of vaccine adverse effect and is observed in about 51–64% of patients with SCID that had not been previously diagnosed^2,3^.

In addition to BCG, other live attenuated vaccines such as rotavirus, oral polio vaccine, measles, mumps, rubella, varicella, and yellow fever vaccines (YFV) are also offered in Brazil^4^. Yellow fever (YF) is an acute febrile infectious disease transmitted by arthropod vectors and caused by a virus of the genus Flavivirus. In recent years, the disease has manifested itself in outbreaks in Brazil, seriously affecting a significant number of patients^4^. As a result, YFV is part of the Brazilian National Immunization Program and offered at 9 months of age^4^. Contraindications to vaccination include infants less than 6 months of age, allergy to a previous dose, allergy to eggs or chicken proteins, and a weakened immune system due to pre-existing disease^4^. Less than 25% of vaccinees develop mild systemic symptoms, which may include headache, myalgia, discomfort at the site of vaccination, or low-grade fever, two to six days after vaccination. Serious adverse events after administration of the YFV are rare and, in most cases, associated with neurological or visceral involvement, the so-called neurologic and viscerotropic disease. Although rare, the vaccine disease can manifest itself more severely in patients with primary immunodeficiency^3,4^.

We present a previously healthy child who was vaccinated with YFV in Brazil and developed severe dissemination of the vaccine virus leading to neurologic and viscerotropic disease. The patient was later diagnosed with SCID.

## CASE REPORT

A 10-month-old female infant sought emergency care presenting fever (>38 °C) for three days and diffuse exanthema. She was a previous healthy child of consanguineous parents with no history of growth delay, illness, or hospitalization. Patient’s parents were also healthy. The mother reported that, 28 days before the onset of symptoms, the child had received yellow fever vaccine (17DD) produced by the Immunobiological Technology Institute (Bio-Manguinhos - Fiocruz). Upon hospital admission, petechial rash on the limbs and hepatosplenomegaly were noted on physical exam. Laboratory tests showed thrombocytopenia, increased serum aminotransferases, and elevated gamma-glutamyl transferase (GGT) and alkaline phosphatase levels (Table 1). Given the clinical and epidemiological picture, serological tests were conducted, including IgM-enzyme-linked immunosorbent assay (ELISA) for leptospirosis, IgM MAC-ELISA for dengue fever, and immunofluorescence assay (IFA) for Brazilian spotted fever, all of them showing negative results. An ultrasound scan of the abdomen was performed, in which no major alterations were identified. Brain CT findings were unremarkable. On the 8^th^ day of hospitalization, she presented hypothermia and inconsistent response to external stimuli, evolving with seizure associated with loss of consciousness. The patient was evaluated by the pediatric neurology team, which recommended new laboratory tests and a brain magnetic resonance imaging (MRI).

**Table 1:** Laboratorial tests of a case report of viscerotropic disease after yellow fever vaccine in an infant patient with severe combined immunodeficiency.

Days of hospitalization	Hb^a^ (mg/dL)	Htc (%)	Platelets (cells/mm^3^)	AST^b^ (U/L)	ALT^c^ (U/L)	AP^d^ (U/L)	GGT^e^ (U/L)	INR^f^	UR^h^	CR^i^
1^st^	10.8	32.2	35,000	1,272	1,096	873	818	1	18.3	0.21
8^th^	9.3	25.5	31,000	1,735	2,442	1,099	789		12.6	0.2
15^th^	10.7	30.2	19,000	869	898	665	745			
22^nd^	10.1	31.0	85,000	795	745	528	877			
35^th^	8.7	26.8	113,000	296	136	331	894	1.2	13	0.15

^a^Haemoglobin; ^b^Aspartate aminotransferase; ^c^Alanine aminotransferase; ^d^Alkaline phosphatase; ^e^Gamma-glutamyl transferase; ^f^International normalized ratio; ^h^Urea; ^i^Creatinine.

Analysis of the cerebrospinal fluid (CSF) returned normal results (cell count: 1 WBC/mm^3^; protein: 56 mg/dL; glucose: 45 mg/dL; red blood cell: 1; Gram stain: negative). MRI showed mild brain volume reduction, also evident in the midbrain, less prominent in the cerebellar hemispheres. Foci of signal alteration were detected in the ponto-mesencephalic transition region and right thalamus, indicating prolonged relaxation time in long TR sequences. No diffusion restriction was found, which suggested a subacute vascular event or an inflammatory disease. The possibility of acute disseminated encephalomyelitis (ADEM) or viral encephalitis was considered, and the patient received pulse-therapy with corticosteroids for five days, without improvement. On the 15^th^ day of hospitalization, the patient still presented symptoms of acute hepatitis and encephalitis, which progressed to respiratory failure and shock. Then, the child was transferred to an intensive care unit (ICU) of a pediatric hospital of reference to continue the investigation.

The possibility of vaccine-associated viscerotropic and neurotropic disease was considered by the infectious disease team since the onset of symptoms followed the first dose of YFV. The patient was tested for antibodies against the yellow fever virus (MAC-ELISA) on serum and CSF samples, showing positive IgM results. (Table 1)

A primary immunodeficiency hypothesis was also considered in view of the lymphopenia and family history of consanguinity. Reverse transcription-polymerase chain reaction (RT-PCR) showed the absence of T-cell excision circles (TRECs). The number of T lymphocyte cells were CD3 T cells: 50 cells/mm^3^, CD3+CD4+: 38 cells/mm^3^ and CD3+CD8+ T cells:7 cells/mm^3^(reference values: CD3: 2,156–5,004 cells/mm^3^, CD4: 1,360–3,066 cells/mm^3^, CD8: 560–1,803 cells/mm^3^). It was not possible to perform CD19 and NK exams. Following the Primary Immune Deficiency Treatment Consortium (PIDTC/2022), the child was diagnosed as having SCID.

After 22 days of hospitalization, due to the insufficient response to corticosteroids, intravenous immunoglobulin (IVIG) 2 g/kg over three days was administered. Despite that, the child remained in severe condition with no response to treatment. After 35 days of hospitalization, she evolved to cardiorespiratory arrest and death. The adverse vaccine events and the patient’s death were later reported by the medical team to the state health department. Autopsy after patient’s death was not performed.

## DISCUSSION

The 17DD YFV is produced from live attenuated virus and is part of the Brazilian Expanded Program of Immunization since 1998^4^. Despite the strong safety profile, reports of rare yellow fever vaccine-associated serious adverse events have been described, including allergic reactions, neurotropic disease, and viscerotropic disease^5^. The mechanisms underlying the development of these events remain unknown. Distinct hypotheses have been postulated, including viral and host features. It is considered that host factors, mainly host immune responses to the vaccine, may represent the major cause. A history of thymus disorder and immunocompromise are considered risk factors that may explain the serious events observed in this study^5^.

Attenuated vaccines are composed of pathogens that are modified to become weakly virulent and can cause a mild, mostly asymptomatic, and self-limiting infection in immunocompetent patients^5^. Infections with live attenuated microorganisms, however, may not be self-limiting in patients with inborn errors of immunity (IEI) and may be the first and most severe presentation in these patients, as demonstrated in the present case^6,7^. Severe infections from live attenuated vaccines have been recognized in patients with well-defined IEI, including BCG, measles, and oral rotavirus vaccines in patients with SCID, as well as oral polio vaccine (OPV) in those with agammaglobulinemia^8^. This is the first description of a severe adverse event to YFV in an infant with SCID that was not previously diagnosed. We highlight that, although she had not received the oral polio vaccine, she had already been vaccinated with other live vaccines, including rotavirus and BCG vaccines, presenting no symptoms suggestive of immunodeficiency until then. Expanding knowledge about inborn errors of immunity and creating strategies that can contribute to early diagnosis, such as the regulation of neonatal screening for SCID, is extremely important to avoid live vaccines and unfavorable outcomes in these children^9^.

Neurological disease associated with YFV is a rare adverse event and occurs in 0.2 cases/100,000 individuals^10^. It appears from one to four weeks after vaccination and is usually a self-limiting disease in healthy individuals^6^. It has recently been suggested that the neurological adverse events are due to the immune response to YFV virus infection rather than the direct action of the virus through the central nervous system^5^. The YFV is contraindicated for infants younger than six months based on the risk of neurotropic disease in these patients, and we found two randomized clinical trials indicating the safety of YFV in infants from 9 months of age onward^11^.

In an analysis of 67 patients with neurotropic disease in Brazil from 1999 to 2005, 50% reported aseptic meningitis in individuals aged older than 15^6,12^. The disease can be caused directly by viral invasion of the central nervous system or by inflammatory and demyelinating reactions, represented by autoimmune manifestations such as acute disseminated encephalomyelitis (ADEM) or Guillain-Barré syndrome^6^. Clinical and laboratory data of the reported case in this study are consistent with the diagnosis of vaccine encephalitis following the Brighton Collaboration diagnostic criteria^13^. The diagnosis of ADEM was excluded upon confirmation of viral infection of the central nervous system via IgM detection^14^. Detection of pathogen-specific IgM-class antibodies in CSF is widely recognized as indicative of viral invasion of the central nervous system and constitutes a relevant indication of causality^15,16^. In contrast to detecting yellow fever IgM in CSF, limited value for diagnosing meningoencephalitis has been found for molecular testing^15^.

Viscerotropic is even rarer than neurotropic disease and occurs in 0.04 cases/100,000 individuals, usually after the first vaccine dose. In these cases, similar symptoms to those of a patient who has wild-type disease happens in a patient recently vaccinated. Thomas^17^, in a literature review, have described 62 cases of viscerotropic disease all coming from developing countries. Our patient met Brighton Collaboration case definition of viscerotropic disease, as she presented major disease manifestations such as aspartate aminotransferase (AST) or alanine transaminase (ALT) levels >3 times the upper limit of normal, platelet disorder, hypotension, and respiratory distress^17^. Since many other diseases in YF areas can mimic aspects of viscerotropic disease, the definitions also emphasize laboratory confirmation. Older age has been described as a risk factor for viscerotropic disease^17^.

Reports of viscerotropic disease associated with neurotropic disease are rare^5^. The Global Alliance for Vaccines and Immunization, in a systematic review of the safety of YFVs found only two adult patients with viscerotropic and neurotropic diseases^17^. More recently, in 2021, another study reported a 19-year-old previously healthy woman who died 14 days after vaccination^16^. It is important to note that this patient also exhibited some abnormalities in her cellular immune response, suggesting the hypothesis that a robust adaptive response and abnormalities in the innate immune system may be involved in the establishment of viscerotropic and neurotropic events following primary vaccination^5^.

According to a 2016 review, only 36 deaths have been recorded from vaccine-associated viscerotropic diseases, most of them happening in older adult patients^17^. Similarly, few reports have described deaths due to the vaccine-associated neurological diseases^17^. More recently, a Brazilian study conducted in 2017 and 2018, during a massive immunization campaign in response to an outbreak, identified two deaths related to vaccine-derived neurotropic disease. Viscerotropic disease was also found in one of these patients^16^. We found no previous reports of deaths in infants with simultaneous involvement of viscerotropic and neurotropic disease.

## CONCLUSION

The unfavorable outcome observed in this case study highlights the importance of neonatal screening for severe combined immunodeficiency disease and clinical suspicion of primary immunodeficiencies in infants who have serious adverse events to live virus vaccines. Despite these events being rare, live vaccines administered in the first year of life can be devastating in children with immune defects.
